# Pathogenesis of Liver Fibrosis and Its TCM Therapeutic Perspectives

**DOI:** 10.1155/2022/5325431

**Published:** 2022-04-28

**Authors:** Yang Nan, HongChan Su, XiaoMei Lian, Juan Wu, SuJie Liu, PingPing Chen, ShuMin Liu

**Affiliations:** ^1^Heilongjiang University of Chinese Medicine, College of Pharmacy, Heilongjiang, Haerbin 150040, China; ^2^Heilongjiang University of Chinese Medicine, Chinese Medicine Research Institute, Heilongjiang, Haerbin 150040, China

## Abstract

Liver fibrosis is a pathological process of abnormal tissue proliferation in the liver caused by various pathogenic factors, which will further develop into cirrhosis or even hepatocellular carcinoma if liver injury is not intervened in time. As a diffuse progressive liver disease, its clinical manifestations are mostly excessive deposition of collagen-rich extracellular matrix resulting in scar formation due to liver injury. Hepatic fibrosis can be caused by hepatitis B and C, fatty liver, alcohol, and rare diseases such as hemochromatosis. As the metabolic center of the body, the liver regulates various vital activities. During the development of fibrosis, it is influenced by many other factors in addition to the central event of hepatic stellate cell activation. Currently, with the increasing understanding of TCM, the advantages of TCM with multiple components, pathways, and targets have been demonstrated. In this review, we will describe the factors influencing liver fibrosis, focusing on the effects of cells, intestinal flora, iron death, signaling pathways, autophagy and angiogenesis on liver fibrosis, and the therapeutic effects of herbal medicine on liver fibrosis.

## 1. Introduction

**Figure 1:**
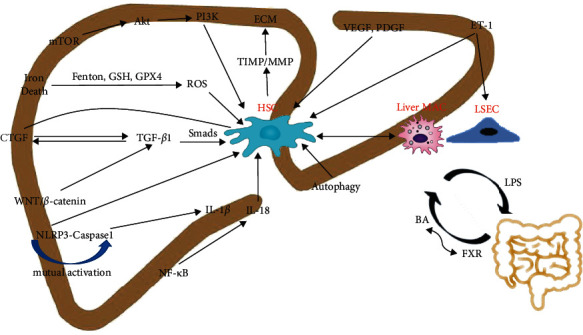
Diagram of pathogenesis associated with liver fibrosis. Intestinal-liver interactions; lipopolysaccharide (LPS); bile acids (BA); nuclear transcription factor receptor (FXR); hepatic stellate cells (HSC); liver macrophages (Liver MAC); liver sinusoidal endothelial cells (LSEC); transforming growth factor (TGF); connective tissue growth factor (CTGF); WNT/*β*-linked protein (WNT/*β*-catenin); NOD-like receptor protein 3 (NLRP3); cysteine aspartate-specific proteinase 1 (caspase-1); nuclear factor-*κ*B (NF-*κ*B); interleukins (IL); glutathione (GSH); glutathione peroxidase 4 (GPX4); reactive oxygen species (ROS); mammalian target of rapamycin (mTOR); protein kinase B (Akt); phosphatidylinositol 3-kinase (PI3K); endothelin-1 (ET-1); vascular endothelial growth factor (VEGF); platelet-derived growth factor (PDGF); TIMP/MMP (tissue inhibitor of metalloproteinases/matrix metalloproteinases); ECM (extracellular matrix).

Liver fibrosis is a wound-healing response when the liver is injured, showing a dynamic process, which can be caused by nonalcoholic steatohepatitis (NASH), nonalcoholic fatty liver disease (NAFLD), and cholestatic liver disease [1]. In addition, liver fibrosis is a determinant of mortality in NASH [2]. Advanced liver fibrosis creates the risk of cirrhosis and hepatocellular carcinoma, which kills approximately 1 million people worldwide each year as a complication of cirrhosis, while hepatocellular carcinoma ranks as the 16th most common cause of death, making the early diagnosis of liver fibrosis crucial [3]. Fibrosis is characterized by the production of myofibroblasts (MFB) that promote scar formation by activated Hepatic stellate cells (HSC), and the synthesis of extracellular matrix (ECM) by both MFB and HSC, and the balance of ECM is regulated by both matrix metalloproteinases (MMP) and tissue inhibitor of metalloproteinases (TIMP) that ultimately target HSC to form fibrosis, so it can be said that the dynamics of ECM regulation process is the process of liver fibrosis formation. In addition, nonparenchymal cells such as macrophages (MAC) and liver sinusoidal endothelial cells (LSEC) are also involved [4]. In addition, various signaling pathways, including transforming growth factor (TGF-*β*), platelet-derived growth factor (PDGF), and nuclear factor-*κ*B (NF-*κ*B), are also key pathways affecting liver fibrosis. Currently, there are still no specific and effective drugs to treat liver fibrosis, but there is increasing evidence that Chinese medicine and natural products provide effective help in the prevention and treatment of liver fibrosis [5, 6]. Therefore, in this paper, a large number of collections were carried out in the PUBMED database to fully understand the main events of liver fibrosis, which provided the possibility for targeted liver fibrosis therapy, as shown in Figure 1.

## 2. Cellular Factors

HSC, MAC, and LSEC all have a dual role in the formation of liver fibrosis. During liver injury, the activation and proliferation of hepatic stellate cells, the increase of Ly6C^hi^ in macrophages, the capillarization of hepatic sinusoidal endothelial cells, and the concomitant secretion of various inflammatory factors all contribute to the formation of fibrosis. The apoptosis of hepatic stellate cells, the increase of Ly6C^lo^ in macrophages, and the normal differentiation of hepatic sinusoidal endothelial cells can make the activated hepatic stellate cells quiescent and even degrade the excess extracellular matrix, which can effectively prevent the development of liver fibrosis. This article will explain how cellular factors are actively activated to cause liver fibrosis.

### 2.1. Hepatic Stellate Cells

Hepatic stellate cells (HSC) are a class of nonparenchymal cells located in the endothelial cells of the hepatic sinusoids and the sinusoidal spaces surrounding the hepatocytes, also known as hepatic lipid storage cells. Under pathological conditions, HSC is converted to an activated state by stimulation of various environmental factors, which is a critical step in the development of liver fibrosis [7, 8]. Chronic liver injury leads to multiple damage-associated molecular patterns (DAMPS) producing a series of cytokines including insulin growth factor (IGF-1) and ET-1 that activate the secretion of HSC. At the same time, long-term stimulation that triggers an inflammatory response combined with LSEC [9] and KC [10] also releases damage factors like IL-6, TNF-*α*, and TGF-*β* to further promote the initiation of HSC activation [11]. Activated HSC themselves also secrete fibrogenic factors such as CTGF and leptin, which in turn enhance the ability to induce proliferation of HSC. In addition, *α*-SMA-containing myofibroblasts (MFB) contribute to the formation of fibrotic scar and synthesize I and III collagen-based ECM [12, 13], and excessive accumulation of ECM activates HSC, creating a positive feedback loop leading to fibrosis formation. In particular, PDGF and VEGF released from platelets are mitogenic mediators of HSC and bind to ECM to enable the already activated HSC to undergo the next step of proliferation [14]. Chemokine-chemokine receptors play a key role in liver fibrosis, especially the C-C motif chemokine receptor 2 (CCR2) activates HSC [15]. The process by which epithelial cells gradually lose their phenotypic characteristics while acquiring mesenchymal cell characteristics is called epithelial-mesenchymal transition (EMT), and the involvement of EMT also mediates the transdifferentiation and fibrosis of HSC [16]. When the Hh signaling pathway derepresses Smo and activates the Gli transcription factor, Gli contains a predicted paired frame 6 (PAX6) binding site in its transcriptional region, which promotes both HSC activation and proliferation [17].

### 2.2. Macrophages

Macrophages are derived from precursor cells in the bone marrow, which are immune cells [18]. Those active in the liver are called liver macrophages (Liver MAC) and mainly include kwashiorkor cells (KCs) and monocyte-derived macrophages (MoMF) [19].

Under pathological conditions, there is a large amount of Ly6C^hi^ from MoMF, which has a proinflammatory properties and can overexpress CCR1 and CCR2 [20], and Ly6C^hi^ can not only release a large number of cellular, inflammatory, and chemokines such as TGF-*β*, PDGF, TNF-*α*, IL-1*β*, and CCL2 [21–24] but can also depend on chemokine aggregation to the site of liver injury. The precursor cells of Ly6C^lo^ are Ly6C^hi^ monocytes, but Ly6C^lo^ has the opposite effect to Ly6C^hi^, where Ly6C^lo^ can downregulate inflammatory factors and increase MMP, promoting the degradation of ECM. Through the study, when the CCR2 gene was knocked down in mice, Ly6C^hi^ was reduced and Ly6C^lo^ quantity was increased in the liver, corresponding to the reduction of HSC activation and some relief of liver fibrosis, indicating that Ly6C^hi^ has proinflammatory and profibrotic properties, while Ly6C^lo^ has anti-inflammatory and antifibrotic properties.

KCs are located in macrophages within the hepatic sinusoids, also known as resident cells [25]. When a liver injury occurs, KCs are activated by DAMPs and pathogen-associated molecular patterns (PAMPs) interacting with Toll-like receptors (TLRs). Activated KC generates various types of mediators of liver fibrosis progression (e.g., TGF-*β*, PDGF, IL-1*β*, MMPs, CCL2, cysteine-3, etc.) and also accelerates the progression of liver fibrosis by activating HSC to produce large amounts of collagen, allowing ECM to settle and aggregate [26].

### 2.3. Liver Sinusoidal Endothelial Cells

Liver sinusoidal endothelial cells (LSEC) are highly specialized endothelial cells with pores on the cell surface and open windows, whose vascular secretory signals regulate liver function and have an important role in maintaining the homeostasis of the hepatic endotrophic environment, which, in turn, is a key factor in the activation of HSC [27, 28]. Differentiated LSEC can maintain HSC in a quiescent form, which can accelerate the regression of liver fibrosis and stop its progression, but the opposite is true for capillary LSEC [29]. When LSEC forms an organized basement membrane and lacks open windows, it is capillary vascularization of LSEC, which then eventually leads to the activation of HSC through the synthesis of factors like TGF-*β* to form fibrosis, fibrosis aggravates LSEC, and LSEC promotes fibrosis, leading to a vicious cycle. The vascular endothelial factor VGEF pathway was found to protect LSEC from opening windows and prevent fibrosis [30]. Capillarization is induced in LSEC if the Gata4 gene is absent, inducing the expression of profibrotic vascular secretory factors, which in turn leads to the possibility of perisinusoidal capillarization or fibrosis and liver lesions [31]. It has been claimed that the addition of bone morphogenetic protein (BMP9) to LSEC in primary culture, as a regulator of the intrahepatic environment, not only prevents the loss of window pores but also integrates the Gata4 gene and restores LSEC differentiation [32]. Furthermore, autophagy affects liver fibrosis by affecting LSEC [33], when the liver is mildly injured, autophagy of LESC enhances sinusoidal endothelial dysfunction (ED) and activates HSC, but, if the liver is too long or more severely injured, autophagy decreases, and ED fails to proceed. Autophagy of LSEC also enables chemokines, inflammatory factors such as C-C chemokine ligand 2 (CCL2), C-C chemokine ligand 2 (CCL5), and interleukin-6 (IL-6) are enhanced, promoting the hepatic inflammatory response and thus liver fibrosis [34].

## 3. Signaling Pathways

### 3.1. TGF-*β*1/Smads Signaling Pathway

Transforming growth factor *β*1 (TGF-*β*1) is the most prominent way to promote fibrosis formation [35], which is through the intracellular Smads signaling pathway [36]. The Smads pathway promotes HSC activation, and inactivated HSC and activated Smad2 and Smad3 induce type I and type III collagen production [37], promote MFB cell proliferation, and increase ECM deposition in the liver [38]. Smad4 inhibits the binding activity of Smad3 to collagen and the aggregation and degradation of ECM in vitro and in vivo. In acute liver injury, Smad7 can compete with receptor-activated Smad2 and Smad3 to bind TGF-*β*1 receptors and reduce ECM production, or Smad7 can interrupt TGF-*β*1 signaling by enhancing the degradation of TGF-*β* receptors. However, after HSC transdifferentiation to MFB, the ability of TGF-*β*1 to induce Smad7 expression decreases, Smad2 and Smad3 are phosphorylated, the TGF-*β*1/Smads signaling pathway is activated, and ECM is secreted in large amounts, accelerating liver fibrosis [39–41]. TGF-*β*1 can also promote liver fibrosis by activating non-Smads pathways, such as MAPK, NF-*κ*B, and PI3K [42]. TGF-*β*1 can induce TIMP-1 expression after signaling to Smad3, while inhibiting MMP-1 expression, making the ratio of TIMP-1 to MMP-1 increase, and promoting liver fibrosis. Smad2 can induce MMP-2 expression, and by knocking down the Smad2 gene, we obtained that TGF-*β*1 through the Smad3 pathway upregulated the expression of TIMP-1 and inhibited the expression of MMP-2, which in turn inhibited the degradation of ECM [43].

### 3.2. WNT/*β*-Catenin Signaling Pathway

Wnt acts as a signaling cascade that transfers signals through cell surface receptors to protein components in the cell. There are two types of this signaling pathway, the nonclassical planar cell polarization pathway that regulates the cytoskeleton and thus cell shape, and Wnt/Ca^2+^ that regulates intracellular Ca^2+^ concentration, and the classical signaling pathway that regulates specific gene transcription, which is dependent on *β*-catenin for further action on downstream factors and ultimately translocates to the nucleus where it binds to the intranuclear transcription factor Tcf/Lef, thereby activating expression of the relevant target gene [44]. The main difference between the two pathways is that the nonclassical pathway operates independently of it, and the classical pathway involves the involvement of *β*-catenin. It is believed that the Wnt/*β*-catenin pathway is now considered to be the main influential pathway involved in the development of liver fibrosis, and *β*-catenin has an important role as a key signaling molecule in this pathway [45]. When Wnt is activated and *β*-catenin is phosphorylated, ECM accumulation leads to HSC activation [46]. The deletion of *β*-catenin leads to TGF-*β* upregulation and oxidative stress [47]. In addition to the apoptotic effects of HSC that can be produced by the inhibition of Wnt and *β*-catenin [48], many substances can influence the homeostasis of the intestinal flora, the progression of cirrhosis, and the activation of inflammatory factors by participating in the WNT/*β*-catenin signaling pathway, which affects the process of fibrosis [49]. This justifies the reliability of using the Wnt/*β*-catenin signaling pathway as an entry point for the treatment of fibrosis. Silencing or inhibition of *β*-catenin, in the Wnt/*β*-catenin signaling pathway, promotes improvement of liver fibrosis or cirrhosis by limiting the contractility of HSC and portal hypertension [50].

### 3.3. Inflammasome (NLRP3) Caspase-1 Signaling Pathway

Inflammasome (NLRP3) is a part of the natural immune system, and it has been shown that all components of NLRP3 are present in the HSC, that multiple functions of the HSC are regulated by NLRP3, and that NLRP3 recognizes the release of damage patterns such as DAMPs and PAMPs caused by liver injury, by recruiting and activating proinflammatory cysteine-containing aspartate proteolytic enzyme 1 caspase-1, so that activated caspase-1 activates the proinflammatory cytokines IL-1*β* and IL-18, leading to the activation of HSC and the occurrence of liver fibrosis [51–53]. IL-1*β* aggregates by recruiting neutrophils, and excess proinflammatory cytokines lead to the activation of reactive oxygen species (ROS), inflammatory cells, and growth factors, which not only further promote activation of inflammatory vesicles but also promote the activation of HSC, leading to liver fibrosis [54]. Signals induced by lipopolysaccharide (LPS) [55] can activate pro-IL-18 and pro-IL-1*β* to activated IL-18 and IL-1*β* via NF-*κ*B, which can then activate liver fibrosis via proinflammatory cytokines, or they can act directly on NLRP3 inflammatory vesicles, which in turn activate its downstream signaling pathway caspase-1 via NLRP3 inflammasomes which in turn will promote proinflammatory processes in the maturation and secretion of the precursors pro-IL-1*β* and pro-IL-18 during the defense process, promoting liver fibrosis formation [42, 56–58]. At the same time, caspase-1 also induces the activation of intracellular NLRP3 inflammatory vesicles, which can lead to a vicious cycle of proinflammatory signaling [59].

### 3.4. Phosphatidylinositol 3-Kinase (PI3K)-Akt Signaling Pathway

Phosphatidylinositol 3-kinase (PI3K) is a class of inositol lipid substance kinases, the most widely studied class of which is I PI3K, with the regulatory subunit p58 and the catalytic subunit p110 as the main targets [60]. Protein kinase B (Akt) is a downstream target of the PI3K signaling pathway and regulates the cell-initiated kinase cascade reaction that allows AKT to be activated by being readily located on the plasma membrane [61] PI3K and Akt are involved in the regulation of various signaling pathways including liver fibrosis in the liver. Studies have found that the autophagy of BDL and CCL4-induced liver fibrosis can be activated to promote liver injury by activating the TGF-*β*1/Smads signaling pathway and inhibiting PI3K/Akt signaling pathway and regulating cross-talk between the two pathways [62]. The initiation of phosphorylation of PI3K, AKT, and even mTOR in the PI3K/AKT signaling pathway can trigger the activation and proliferation of HSC and affect the production of liver fibrosis [63]. It is noteworthy that mTOR, as a downstream signaling molecule of AKT, also plays an important role in this pathway [64]. The expression of mTOR acts on the PI3K/Akt signaling pathway, induces HSC proliferation, induces the expression of *α*-SMA and other fibrogenic factors, and induces ECM synthesis [65].

### 3.5. Nuclear Factor-*κ*B Signaling Pathway

Nuclear factor-*κ*B (NF-*κ*B) is a transcriptional regulator of greater interest in liver fibrosis and consists of a heterodimer of RelA (p65) and p50 subunits. When a sustained liver injury occurs, it activates HSC and KC, which in turn releases proinflammatory, chemotactic, and cytokines, causing the recruitment of various factors to the damaged sites of liver inflammation and stimulating NF-*κ*B, which in turn stimulates the production of each factor upon NF-*κ*B activation, thus creating a positive feedback between NF-*κ*B and inflammatory factors [66]. Studies have shown that the NF-*κ*B pathway is a key factor in HSC activation and proliferation, and it inhibits apoptosis and promotes HSC activation. When the expression of NF-*κ*B inhibitor protein *α* (I*κ*B*α*) increased, the Bax/Bcl-2 ratio rises, the NF-*κ*B signaling pathway is inhibited, and the process of liver fibrosis is slowed down. In contrast, when KC is activated, the expression of NF-*κ*B-p65 is significantly increased, the activity of ATL and AST is also significantly increased, and the progression of fibrosis is significantly accelerated [67, 68]. Through experiments [69], the NF-*κ*B signaling pathway can lead to liver fibrosis through miR-378, as miR-378 leads to the development of NASH and liver fibrosis by promoting the activation of the NF*κ*B-TNF*α* axis.

### 3.6. CTGF Signaling

Connective tissue growth factor (CTGF) belongs to a signal in the Hippo signaling pathway and is closely associated with various pathways such as TGF-*β*, Ras, MEK, ERK, WNT, AKT, and MAPK [70–74]. Through research [75], activated HSC secretes CTGF, which in turn promotes HSC activation and migration and upregulates type I collagen and *α*-SMA and activated HSC differentiates into MFB, which also secretes a large number of collagen fibers, resulting in a large amount of ECM deposition, and the above process can be analyzed as positive feedback between CTGF and HSC. CTGF may be a downstream response element of the TGF-*β*1 pathway and mediates some of the active effects of the TGF-*β*1 pathway, which allows fibrosis to occur, while CTGF has a role in maintaining fibrosis by activating a series of transduction pathways that induce MFB proliferation and ECM synthesis and promote the formation of liver fibrosis [76, 77].

## 4. Intestinal Flora

### 4.1. PAMP

In addition to the intestinal flora, which can help the body to digest and absorb and regulate metabolism, its components and functions and the alteration of the intestinal barrier can directly or indirectly affect the formation of liver disease, and in turn, the occurrence of various liver diseases can affect the stability of the intestinal flora [78, 79]. This is because the liver and the intestine are connected by the portal vein, bile passes through the liver to act on the intestine, nutrients from the intestine flow into the portal circulation to reach the liver, and the two interact to form a feeder loop called the enterohepatic axis [80, 81]. Amid this loop, if there is an imbalance in intestinal homeostasis or translocation between microbial components, etc., called PAMP, hepatocytes such as KC and HSC and immune receptors in the lamina propria of the intestine recognize this pattern, leading to an inflammatory response and thus inducing fibrosis [82]. Thus, it can be said that intestinal flora disorders induce liver fibrosis.

### 4.2. Lipopolysaccharide

When intestinal permeability is disrupted or the microbial composition of the gut is altered, a series of metabolic toxins are produced, such as LPS, an endotoxin that further affects liver function, mainly by triggering an inflammatory response, via the enterohepatic axis. In binding to Toll4, a Toll-like receptor, or mediated through kupffer cells, inflammatory factors are produced into the portal circulation [83]. Naihua Hu et al. [84] demonstrated that different concentrations of LPS had an inducing effect in a model of inflammation and fibrosis in LX2 cells, resulting in enhanced expression of inflammatory factors such as IL-6, IL-1*β*, and TNF-*α*. In addition, LPS was able to regulate the expression of the NF-kB signaling pathway and MAPK proteins, including the JNK and p38 pathways associated with the activation of inflammatory mediators, with the consequent production of inflammatory factors that induce liver fibrosis formation [85].

### 4.3. FXR

Nuclear transcription factor receptor (FXR) and bile acids (BA) also interfere with fibrogenesis in terms of intestinal flora; BA is synthesized by the liver and acts in the intestine to produce secondary bile acids that inhibit FXR, which together with BA regulate the homeostasis of intestinal flora. When elevated concentrations of BA induce physiological activation and proliferation of HSC leading to pathological lesions of liver fibrosis, FXR can reverse this phenomenon by maintaining low concentrations of bile in hepatocytes. In addition, it prevents liver injury, which triggers liver fibrosis when FXR is inhibited [86, 87]. In addition, FXR can regulate the inflammatory response activated by BA in concert with LPS, mainly because FXR interconnects with NLRP3 or caspase-1 to produce nonpositive regulation of NLRP3 action [88].

## 5. Autophagy

When HSC is normal, its cytoplasm contains a large number of lipid droplets [89]. When the liver is damaged and the HSC is activated, the lipid droplet content in the cytoplasm decreases, and as the HSC is converted to MFB, the autophagic flux increases, and when inhibited by the autophagy inhibitor bafilomycin A1, the lipid droplet content normalizes and the HSC returns to normal; i.e., the increased autophagic content is associated with HSC activation [90, 91]. Because autophagy lysosomes degrade abnormal proteins, etc., eliminating intracellular metabolic wastes and improving HSC survival and the substances recovered by autophagy provide energy and nutrients to HSC [92]. Studies have shown that autophagy is dependent on ROS, mTOR, and IL-7, and another factor in the process of liver fibrosis [93, 94]. When damage occurs in hepatocytes, it stimulates the production of large amounts of ROS in the liver, and the accumulation of excessive ROS can exceed the normal range of the antioxidant system in the body, allowing the occurrence of oxidative stress that causes abnormal fibrosis in the liver, while the activation of mTOR accelerates liver fibrosis when the organism is in a state of nutritional deficiency [95]. Autophagy can also protect the liver by degrading abnormal metabolites to prevent cellular damage or generate excessive autophagy leading to cellular scorching and the inability of HSC to survive [96, 97].

## 6. Iron Death

Iron death can occur through the Fenton reaction that generates a large amount of ROS that accumulate in the body and cannot be metabolized in time, leading to excessive oxidative stress, promoting lipid peroxidation and oxidative damage to the cell membrane, which in turn leads to cell death [98–100]. When the body lacks glutathione (GSH) also causes excessive accumulation of ROS and iron death, and when glutathione peroxidase 4 (GPX4) activity decreases, polyunsaturated fatty acids are produced in the body to undergo lipid peroxidation and cannot be metabolized by GPX4, resulting in cell death by intracellular hoarding [101, 102].

In the liver, HSC contains iron ions, and the development of liver fibrosis is closely related to iron ions and lipid peroxidation in HSC, and the progression of liver fibrosis can be regulated by iron death. When hepatic iron concentration (HIC) exceeds the normal level, HSC function is abnormal, and in more severe cases, it progresses to cirrhosis. Iron death in HSC leads to an increase in *α*-SMA and type I collagen, allowing ECM deposition, or increased production of proinflammatory factors through TGF-*β* and NF-*κ*B signaling pathways, resulting in liver fibrosis. Liver fibrosis due to iron death also occurs mostly in hepatocytes and macrophages, which can likewise lead to an inflammatory response through the overproduction of HIC and inflammatory cells, activating HSC and producing liver fibrosis [103]. Through studies [104], the therapeutic effect of MgIG on liver fibrosis is produced by promoting the accumulation of iron and lipid peroxides, and the complete disappearance of the antifibrosis effect of MgIG when the inhibitor Fer-1 inhibits iron death in liver fibrosis also confirms the strong association between iron death and liver fibrosis.

## 7. Angiogenesis

Angiogenesis is an important factor contributing to liver fibrosis and is essential for the repair of liver injury [105]. LSEC and HSC express a range of proangiogenic cytokines, including vascular endothelial growth factor (VEGF), endothelin-1 (ET-1), and platelet-derived growth factor (PDGF), with downregulation of VEGF expression and upregulation of ET-1 expression indicating that LSEC undergoes capillarization [33].

### 7.1. VEGF

HSC can promote the expression of VEGF and even the receptor of VEGF, resulting in angiogenesis-induced fibrosis, and VEGF has a better ability to regulate angiogenesis than other cytokines, and, in terms of intestinal inflammation and monocyte infiltration, VEGF also produces effects leading to fibrosis [105]. It was shown that, after histochemical scoring, VEGF can be used as a predictor for the study of fibrosis progression [106]. In addition, VEGF is known to have the ability to alleviate liver fibrosis in bone marrow mesenchymal stem cells (BMSC), and it has been reported that VEGF can increase the permeability of sinusoidal endothelial cells and can affect changes in tissue collagen, a pathway in which BMSC can be better expressed [107].

### 7.2. ET-1

ET-1 is caused by the massive accumulation of extracellular matrix ECM activated by HSC and also stimulates proliferation and collagen synthesis by inducing contraction of activated HSC, which can act on LSEC in addition to HSC [108], and ET-1 can reduce the size and number of window pores in LSEC [30], which in turn can affect liver microcirculation. In CCL4-induced liver fibrosis, ET-1 expression was increased, suggesting that ET-1 could be a promising marker [109]. Not only that, ET-1 acts as a mitogen of smooth muscle vessels with its receptors ET_A_R and ET_B_R to regulate vasoconstriction and diastole. When ET-1 is upregulated in the expression of HSC, ETR is also elevated, leading to increased hepatic sinusoidal resistance and producing portal hypertension, which suggests an exacerbation of fibrosis [110].

### 7.3. PDGF

PDGFs have five isoforms and two receptors, PDGFR-*α* and PDGFR-*β*. PDGFR-*α* has more affinity than PDGFR-*β* and binds more easily to other signaling molecules, such as Ras, MEK, and the extracellular signal-regulated kinase ERK, which are involved in the regulation of fibrogenesis [111, 112]. PDGFR-*β* expression shows an upregulation trend with HSC activation and PDGFR-*β* acts as the most promising proliferation mediator for HSC, inducing further HSC proliferation [113]. Liver sinusoidal endothelial cell vascularization increases liver permeability due to loss of PDGFR-*β* activity [30], and most liver samples from patients with liver fibrosis show increased levels of PDGFR-*β* expression, while PDGFR-*β* is a key pathway to induce HSC activation and proliferation [114].

## 8. TCM Therapeutic Perspectives

So far, the treatment effect of liver fibrosis in Western medicine has progressed slowly, except for surgical treatments such as liver resection; there is no specific curative medicine that can have a good treatment effect on liver fibrosis without complications and adverse reactions. Although the term “liver fibrosis” does not exist in traditional Chinese medicine, it has a long history and profound sources, and the concept of “ruffian, lumps, and accumulation” has been used for a long time, and the modern concept of liver fibrosis belongs to this concept [115]. Moreover, many TCM have the advantages of multicomponent, multipathway, and multitarget, with antioxidant, anti-inflammatory, anticancer, and hepatoprotective effects, and this feature can be fully reflected in liver fibrosis.

Through studies [116–119], tetramethylpyrazine can increase the MMP/TIMP-1 ratio and accelerate the degradation of ECM with antifibrotic effects, block the pathway of TGF-*β*1 and Nrf2/*β*-linked protein, inhibit the activation and migration of HSC, increase the storage of lipid droplets within HSC, or exert antifibrotic effects through cellular autophagy, with anti-inflammatory and antioxidant effects, etc., or inhibit hepatic fibrosis by reducing oxidative stress. *Chrysanthemum* [120, 121] can reverse liver fibrosis, mainly by inhibiting the TGF-*β*1 signaling pathway and thus reducing the value-added activation of HSC. Evodiamine [122] was able to reduce IL-6, TNF-*α*, and types I and III collagen expression, inhibit the TGF-*β*1/Smads signaling pathway, and attenuate liver fibrosis. Breviscapin [123] reduces inflammatory factors by killing TLR4/NF-*κ*B signaling pathway, which can reduce liver fibrosis by decreasing apoptotic response, blocking oxidative stress, and inhibiting inflammation. Curcumin, a major component of turmeric, activates PPAR-*α* signaling, inhibits PI3K/Akt signaling, enhances cellular autophagy, and thus inhibits ECM production [89, 124] and also reduces lipid peroxidation to prevent liver fibrosis. *α*-SMA, a marker of HSC activation, was significantly reduced in expression after treatment with the flavonoid baicalin and reversed the effect of PDGF-BB on promoting the ability of HSC-T6 cells to promote activation [125, 126]. In contrast, *Scutellaria baicalensis* decoction has a preventive effect on liver injury, which may be related to the involvement in the metabolism of some tryptophan to reduce oxidative stress and inhibit collagen regeneration [127]. Emodin extracted from various anthraquinones further affects the fibrotic process by inhibiting epithelial-mesenchymal transition (EMT), reducing Ly6C^hi^ macrophage infiltration Ly6C^hi^ [128], mediating the p53 signaling pathway to induce HSC apoptosis and the TLR4 pathway to slow down inflammation production and lipid deposition in NAFLD models [129, 130]. Moreover, all three can mediate the NF-kB pathway, inhibit the TGF-*β* and the levels of inflammatory factors such as TNF-*α*, and antagonize the profibrotic effects of the inflammatory response [129–135]. Studies have shown that ursolic acid (UA) can regulate EMT or MFB via Rho [136, 137], improve the integrity of the intestinal barrier, reduce intestinal flora disorders [138, 139], and modulate the NOX4/NLRP3 inflammatory vesicle signaling pathway to attenuate liver fibrosis [140].

Yinchenhao decoction [115], whose main components are *Artemisia capillaris* Thunb., *Gardenia jasminoides*, and rhubarb, is one of the classical herbal formulas that can effectively repair liver tissues and cure hepatic fibrosis. Studies have shown [141, 142] that the protective effect of Yinchenhao decoction on liver fibrosis is closely related to PI3K-Akt, TNF, and MAPK signaling pathways and the components of the decoction such as aloe vera emodin, rheinic acid, kaempferol, and quercetin are important components for liver protection, and they not only have therapeutic effects on liver injury caused by CCL4 but also improve fatty liver and inhibit cirrhosis, as well as improving liver fibrosis by inhibiting HSC proliferation and activation. In addition, gardenia glycosides [143], the main component of Yinchenhao decoction, can inhibit TGF-*β*/Smad and ERK-MAPK pathways to inhibit TGF-*β*1-induced EMT and also inhibit type I collagen-induced by the TGF-*β*1 pathway to protect the liver and inhibit the development of liver fibrosis. Fuzheng Huayu capsule (FZHYC), as a Chinese medicine preparation approved by the State Food and Drug Administration (SPDA) of China and the Food and Drug Administration (FDA) of the United States [144], is effective in improving fibrosis or cirrhosis including hepatitis B with liver and kidney deficiency and blood stasis blockage [145]. The expression of HBV liver fibrosis was adjusted by inhibiting HSC activation by altering the number or function of CD4T cells in T lymphocytes [146]. Another study noted that *a*-SMA was significantly inhibited in HSC regulated by the FZHYC action JNK pathway [144]. In addition to this, inhibition of NF-kB signaling pathway, lipid peroxidation, and reduction of HA and collagen content in fibrotic patients help to reach the efficacy of FZHYC in activating blood and removing blood stasis and supporting the deficiency [147–149].

## 9. Summary

In this paper, we mainly elaborate on liver fibrosis from cytokines and various related signaling pathways and then discuss the relationship with liver fibrosis from important aspects such as intestinal flora, autophagy, iron death, and angiogenesis and elaborate the role and mechanism in the process of liver fibrosis. We found that the influence on the onset and regression of liver fibrosis through various pathways cannot be unilateral; it must be multifactorial, multifaceted, and multiple pathways interacting and cooperating to make changes in liver fibrosis. Finally, the research on the treatment of liver fibrosis from the aspect of Chinese herbal medicine is mainly discussed, and it is found that, in experiments, various types of Chinese herbal medicine and compounding play a good therapeutic effect on the suppression of liver fibrosis with fewer side effects, but still need to be used carefully according to the changes of the disease to avoid aggravating liver fibrosis [150]. Moreover, microRNA, Hedgehog (Hh) signaling pathway, PPAR nuclear receptor signaling pathway, and hepatic lymphocytes also have a certain influence on the formation of liver fibrosis. So far, there are no specific drugs for clinical treatment in either Western or Chinese medicine, and compared to the singularity of research on the development of Western drugs, the treatment of liver fibrosis by Chinese medicine and compound prescriptions is based on a holistic approach, regulating all aspects of the body, providing ideas for the treatment of fibrosis and suggesting the great potential of Chinese medicine [151].
